# Socioeconomic disparities associated with mortality in patients hospitalized for COVID-19 in Colombia

**DOI:** 10.3389/fpubh.2023.1139379

**Published:** 2023-03-29

**Authors:** Oscar Ignacio Mendoza Cardozo, Juan Pablo Pérez Bedoya, Lina Marcela Ruiz Galvis, Carlos Andrés Pérez Aguirre, Boris Anghelo Rodríguez Rey, Noël Christopher Barengo, Johnatan Cardona Jiménez, Paula Andrea Díaz Valencia

**Affiliations:** ^1^National Faculty of Public Health, Epidemiology Group, University of Antioquia, Medellín, Colombia; ^2^Escuela de Estadística, Universidad Nacional de Colombia, Sede Medellín, Colombia; ^3^Institute of Physics, University of Antioquia, Medellin, Colombia; ^4^Herbert Wertheim College of Medicine and Robert Stempel College of Public Health & Social Work, Florida International University, Miami, FL, United States; ^5^Faculty of Medicine, Riga Stradins University, Riga, Latvia

**Keywords:** poverty, socioeconomic factors, health disparities, hospitalization, in-hospital mortality, COVID-19

## Abstract

Socioeconomic disparities play an important role in the development of severe clinical outcomes including deaths from COVID-19. However, the current scientific evidence in regard the association between measures of poverty and COVID-19 mortality in hospitalized patients is scant. The objective of this study was to investigate whether there is an association between the Colombian Multidimensional Poverty Index (CMPI) and mortality from COVID-19 in hospitalized patients in Colombia from May 1, 2020 to August 15, 2021. This was an ecological study using individual data on hospitalized patients from the National Institute of Health of Colombia (INS), and municipal level data from the High-Cost Account and the National Administrative Department of Statistics. The main outcome variable was mortality due to COVID-19. The main exposure variable was the CMPI that ranges from 0 to 100% and was categorized into five levels: (i) level I (0%−20%), (ii) level II (20%−40%), (iii) level III (40%−60%), (iv) level IV (60%−80%); and (v) level V (80%−100%). The higher the level, the higher the level of multidimensional poverty. A Bayesian multilevel logistic regression model was applied to estimate Odds Ratio (OR) and their corresponding 95% credible intervals (CI). In addition, a subgroup analysis was performed according to the epidemiological COVID-19 waves using the same model. The odds for dying from COVID-19 was 1.46 (95% CI 1.4–1.53) for level II, 1.41 (95% CI 1.33–1.49) for level III and 1.70 (95% CI 1.54–1.89) for level IV hospitalized COVID-19 patients compared with the least poor patients (CMPI level I). In addition, age and male sex also increased mortality in COVID-19 hospitalized patients. Patients between 26 and 50 years-of-age had 4.17-fold increased odds (95% CI 4.07–4.3) of death compared with younger than 26-years-old patients. The corresponding for 51–75 years-old patients and those above the age of 75 years were 9.17 (95% CI 8.93–9.41) and 17.1 (95% CI 16.63–17.56), respectively. Finally, the odds of death from COVID-19 in hospitalized patients gradually decreased as the pandemic evolved. In conclusion, socioeconomic disparities were a major risk factor for mortality in patients hospitalized for COVID-19 in Colombia.

## 1. Introduction

Endemic infectious diseases such as COVID-19 have had an big impact on the life of the global population throughout history (1). COVID-19 has caused ~633,513,131 million infections and more than 6,603,426 registered deaths as of November 2022 around the world (2). In Colombia, the first case was confirmed in March 2020 and since then 6,310,332 cases and 141,850 deaths have been reported by November 2022. A higher number of deaths was reported in over 50-years-old patients (3). An estimated 10%−20% of all COVID-19 patients developed severe clinical complications including death (4–6). Approximately, 40% of COVID-19 patients admitted to the Intensive Care Unit died (1, 5, 6).

Both genetic factors and social determinants such as economic and educational level, food safety, physical and environmental exposures, among others have been associated with adverse health outcomes (7). Their interaction may lead to increased population vulnerability. It is estimated that the contribution of social determinants in health outcomes can be up to 40% (8). For example, diseases transmitted by food and water, have shown to lead to worse survival conditions in socially disadvantaged children (9).

Inequities in social determinants of health may lead to different unfavorable health outcomes in COVID-19 patients (10–14). Current scientific evidence indicated that the development of severe complications, including death, in COVID-19 patients have been associated with biological, social, and economic factors (12, 15). Poverty and the physical environment have shown to be determining factors in the progress of the disease (11–13). The most vulnerable patient population have been the older adults, the poor and those with the certain comorbidities such as obesity, diabetes and cardiovascular diseases (10, 14).

Colombia is considered as one of the countries with the highest levels of economic inequalities in the world. Access to employment, education and a good quality of life has been one of the lowest in Latin America and may have been associated with severe complications in COVID-19 patients (16). However, the current scientific evidence in regard the association between measures of poverty and COVID-19 mortality in hospitalized patients is scant.

The objective of this study was to investigate whether there is an association between the Colombian Multidimensional Poverty Index (CMPI) and mortality from COVID-19 in hospitalized patients in Colombia from May 1, 2020 to August 15, 2021. We hypothesized that hospitalized patients with CMPI score >20% had a higher risk of dying, compared with patients with CMPI between below 20%.

## 2. Methods

### 2.1. Study design and population

This was an ecological study using data of hospitalized patients who have recovered or died from COVID-19 in Colombia between May 1, 2020, and August 15, 2021. The data was obtained from various publicly available de-identified data bases from different sources. The microdata of patients hospitalized for COVID-19 and their sociodemographic characteristics (age and sex) were retrieved from the National Health Institute (3). Additionally, the data on the prevalence of comorbidities at the municipal level were retrieved from the High Cost Account (CAC in Spanish) (17). Finally, demographic data and CMPI were taken from the National Administrative Department of Statistics of Colombia (DANE in Spanish) at the municipal level (18).

### 2.2. Variables

The main outcome variable in this study was mortality from COVID-19. The main independent variable was the Colombian Multidimensional Poverty Index (CMPI). The CMPI is composed of five dimensions (education of household members, childhood and youth conditions, health, employment, and access to household utilities and living conditions) (19). The CMPI uses the multidimensional poverty measurement methodology proposed by Alkire et al. (20) and, hence, identifies as multidimensionally poor, households that experience a greater number of considered deprivations. The CMPI also embodies a standard of living notion that considers household deprivations as constitutive elements to describe the lack of a minimum standard of living. Additionally, it uses a nested weighting structure, where each dimension is equally weighted, as is each indicator within each dimension. Households that had a deprivation in at least 33.3% of the indicators were considered as poor (20). The CMPI is a percentage of the population with deprivation in each municipality, which indicates that the higher the proportion, the greater the multidimensional poverty in that region. The National Administrative Department of Statistics of Colombia (DANE) collected the data to generate the CMPI (21). The five categories of the CMPI defined by DANE were used. The categories were as follows: (i) level from 0 to 20%, (ii) level II from 20 to 40%, (iii) level III from 40 to 60%, (iv) level IV from 60 to 80%; and (v) level V from 80 to 100% (21). Municipalities with a CMPI <20% were considered as those with the lowest proportion of multidimensional poverty and, thus, used as reference category in the analysis. Information on chronic diseases used data from 2020 obtained at the municipal level from the High-Cost Account publicly available data base. The information retrieved were prevalence of hypertension, prevalence of diabetes mellitus, prevalence of chronic kidney disease and rate of general invasive cancer. In addition, the COVID-19 infection rate and population density for each municipality of patients with COVID-19 were included.

Whereas information on mortality age and sex were obtained at the individual level. Other variables, including the CMPI, were retrieved at the municipal level (grouped) as common in ecological studies.

### 2.3. Statistical analysis

A univariate descriptive analysis was performed, where the quantitative variables are described using the median and the interquartile range (IQR). Information on categorical variables is presented as frequencies. A bivariate and inferential descriptive analysis was performed based on the outcome death (yes or no) using the Chi-square and Mann–Whitney *U*-tests, with *p*-values <0.05 being considered as statistically significant. Finally, a Bayesian multilevel logistic model (see Supplementary material) was applied to estimate the Odds Ratio (OR) and their corresponding 95% credible intervals (CI). In addition, a subgroup analysis was carried out by epidemiological waves defining the time windows of each one (Supplementary Figure S1) using the Bayesian multilevel logistic model of the R BRMS package (22).

### 2.4. Ethical considerations

This study was classified as a risk-free investigation, according to resolution number 8430/1993 of Ministry of Health of Colombia. All data were de-identified.

## 3. Results

**Figure 1:**
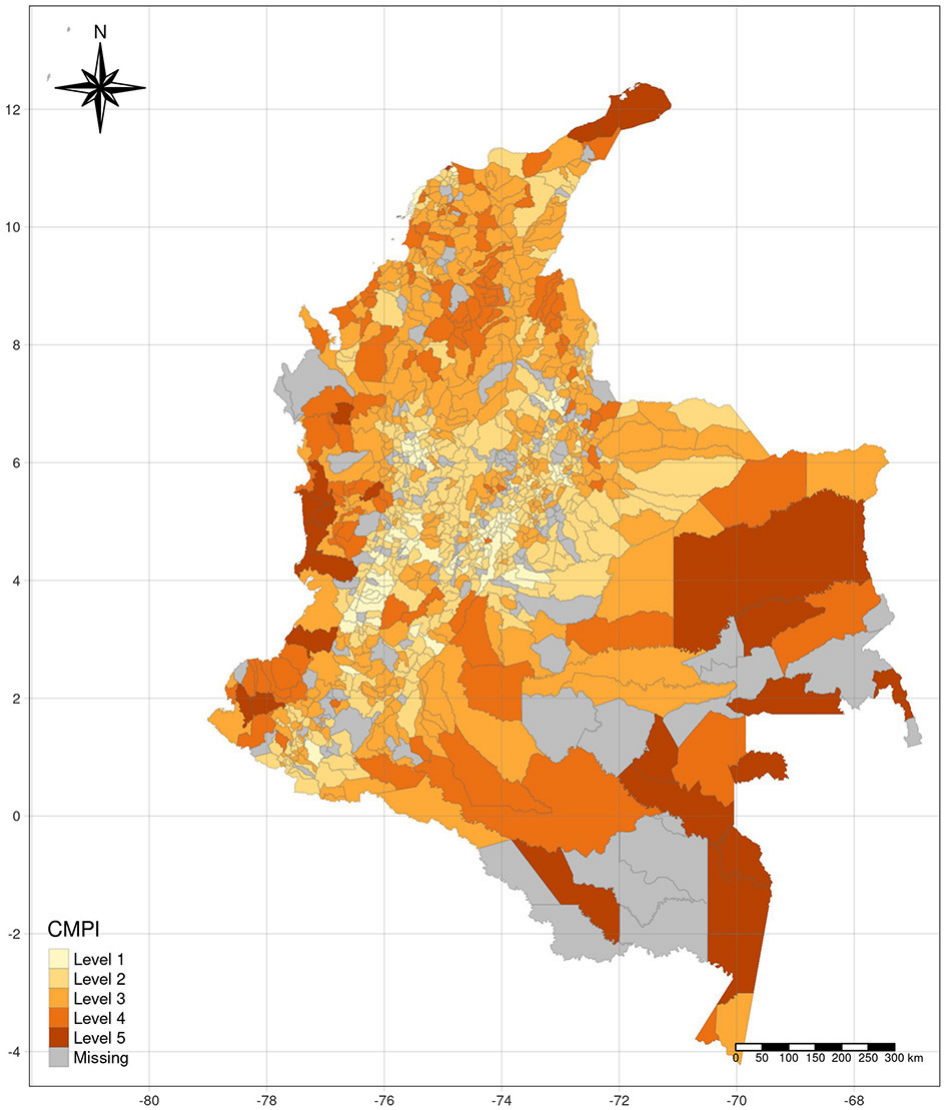
CPMI levels in Colombian municipalities, where notification of patients hospitalized for COVID-19 was made during 2020–2021.

During May 1, 2020, and August 15, 2021, a total of 190,485 patients were hospitalized due to COVID-19 in Colombia of which 45% were women. Their median age was 55 years (IQR 38–68 years). Colombia consists of 1,123 municipalities of which 964 reported patients hospitalized data for COVID-19 in their territories during the observation period selected for this study. The CMPI of each municipality is presented in Figure 1. Overall, 10.68% had a CMPI below 20% (level I). The corresponding percentages for level II, III, IV, and V were 37.66, 37.45, 11.38, and 2.39%, respectively. The gray areas on the map were territories without information on hospitalized patients.

Table 1 shows several characteristics among deceased and non-deceased patients hospitalized for COVID-19 in Colombia. In total, 24% of all hospitalized patients died. CMPI was differently distributed according to mortality. Among those who died, ~10% had a CMPI of at least 40%. The corresponding CMPI in those who survived was 7.5%. In addition, the highest proportion of deaths were found in those over 75 years of age (35%). Likewise, 61% of COVID-19 patients who died were men.

**Table 1:** Sociodemographic factors, socioeconomic characteristics and chronic disease prevalence among deceased and non-deceased patients hospitalized for COVID-19.

	**Death**		**P-value**
	**Yes** ***n*** **(%)**	**No** ***n*** **(%)**	
**CMPI**			
Level I	31,088 (68.88%)	109,872 (75.59%)	<0.05
Level II	9,503 (21.06%)	24,158(16.62%)	
Level III	3,644 (8.07%)	9,141 (6.29%)	
Level IV	813 (1.80%)	1,933 (1.33%)	
Level V	83 (0.18%)	250 (0.17)	
**Age (years)**			
Age (0–25)	5,853 (12.97%)	75,880 (52.20%)	<0.05
Age (26–50)	11,456 (25.38%)	37,553 (25.84%)	
Age (51–75)	11,898 (26.36%)	18,281 (12.58%)	
Age (>75)	15,924 (35.28%)	13,640 (9.38%)	
**Sex**			
Female	27,753 (61.49%)	77,555 (53.36%)	<0.05
Male	17,378 (38.51%)	67,799 (46.64%)	
	**Median (IQR)**	**Median (IQR)**	
Contagion rate	0.11 (0.06–0.12)	0.11 (0.09–0.12)	<0.05
Population density	3,068 (241–4,774)	4,008 (357–4,774)	<0.05
Prevalence of hypertension	10.55 (8.72–11.43)	10.55 (9.50–11.43)	<0.05
Prevalence of diabetes	3.46 (2.88–4.12)	3.46 (3.02–3.95)	<0.05
Prevalence of chronic kidney disease	2.14 (1.49–2.64)	2.38 (1.77–2.64)	<0.05
Prevalence of cancer per 100,000	631 (198–1,516)	665 (219–1,516)	<0.05

IQR, interquartile range.

**Figure 2:**
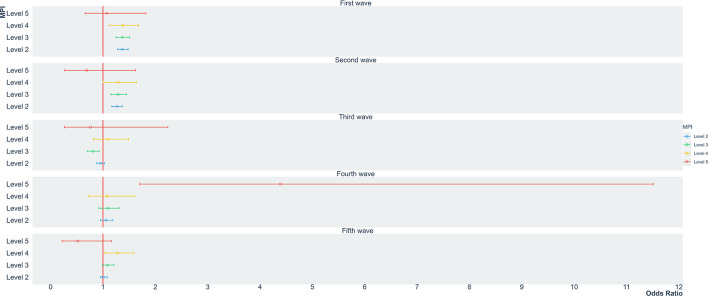
Odds ratio of death according to CMPI levels in patients hospitalized for COVID-19 in Colombia, by COVID-19 waves: First wave: May 1, 2020 to September 9, 2020; Second wave: September 10, 2020 to November 15, 2020; Third wave: November 16, 2020 to February 19, 2021; Fourth wave: February 20, 2021 to May 1, 2021; Fifth wave: May 2, 2021 to August 15, 2021.

The odds for dying from COVID-19 was 1.46 (95% CI 1.40–1.53) for level II, 1.41 (95% CI 1.33–1.49) for level III and 1.70 (95% CI 1.54–1.89) for level IV compared with hospitalized patients with a level I CMPI score (Table 2). In addition, age also increased mortality in COVID-19 hospitalized patients. Patients between 26 and equal to or more than 75 years-of-age had probabilities between 4.07 to 17.56 of death compared with younger than 26-years-old patients. Moreover, men had a statistically significant higher odds of dying compared with women (OR 1.47; 95% CI 1.44–1.51). Figure 2 presents the OR of mortality in patients hospitalized for COVID-19 for each CMPI according to the five epidemiological waves identified between May 1, 2020, and August 15. During the first epidemiological wave, patients above a CMPI of 20% had increased odds of death compared with those below a CMPI below 20%. Whereas the OR of death for those with level II CMPI were 1.37 (95% CI 1.28–1.48), the corresponding odds of those with level III and IV CMPI were 1.37 (95% CI 1.25–1.51) and 1.38 (95% CI 1.13–1.68). A similar pattern was found during the second epidemiological wave were patients with CMPI above 20% were at higher likelihood of dying compared with level I CMPI hospitalized COVID-19 patients. The odds of death were 1.27–1.29-fold higher in level II-IV CMPI patients compared level I patients. No statistically significant association between CMPI and death was found during the third epidemiological wave in Colombia. However, during the fourth epidemiological wave, patients in the poorest CMPI level had 4.39-times higher odds of dying (95% CI 1.70–11.51) compared with those with a CMPI below 20%. Finally, during the fifth COVID-19 wave, only hospitalized patients with a CMPI between 60 and 80% had increased mortality (OR 1.28; 95% CI 1.03–1.59).

**Table 2:** Adjusted associations between multidimensional poverty measures and mortality from COVID-19 in hospitalized patients, adjusted for different covariates.

**Variable**	**OR**	**95% CI**
**CMPI**		
Level I	Ref.	Ref.
Level II	1.46	1.40–1.53
Level III	1.41	1.33–1.49
Level IV	1.70	1.54–1.89
Level V	0.96	0.70–1.31
**Age (years)**		
Age (0–25)	Ref.	Ref.
Age (26–50)	4.17	4.07–4.30
Age (51–75)	9.17	8.93–9.41
Age (>75)	17.10	16.63–17.56
**Sex**		
Female	Ref.	Ref.
Male	1.47	1.44–1.51
Contagion rate	0.29	0.18–0.47
Population density	1	1.00–1.00
Prevalence of hypertension	0.97	0.97–0.98
Prevalence of diabetes	1.11	1.09–1.13
Prevalence of chronic kidney disease	0.90	0.88–0.91
Prevalence of cancer	1	1.00–1.00

OR, odds ratio; CI, credible intervals; Ref., reference category.

## 4. Discussion

Our study revealed that the CMPI was associated with increased mortality in hospitalize COVID-19 patients in Colombia. Patients with a higher CMPI index had a higher likelihood of death compared with those of the lowest poverty level. Moreover, these socio-economic disparities were more pronounced during the first two epidemiological COVID-19 waves than in the later ones.

In general, our data is in line with those reported in other countries such as Spain, Brazil and Germany revealing that socioeconomic disparities in COVID-19 patients were a risk factor of mortality (23–27). However, most of the previous studies were conducted in the general population and not in hospitalized patients. Only the studies by Rodrigues et al. and Hu et al. included hospital patients (15, 28). Additionally, there are only few studies that assessed composite indices related to socioeconomic disparities as predictors of severe complications due to COVID-19 in hospitalized patients (28).

Thus, in the Colombian population, hospitalized patients with a higher CMPI had a higher likelihood of dying compared with the least poor patient segment. This is in agreement with the data reported by Rodrigues et al. (15), reveling that patients hospitalized due to COVD-19 living in poorer economic conditions had a 2.45 times more risk of dying. In contrast to our findings, Hu et al. (28) found that residing in much more deprived neighborhoods increased the risk of hospital mortality from COVID-19 1.74 times (95% CI 1.13–2.67), regardless of race. However, they used a different composite score, the Area Deprivation Index, to measure poverty.

We did not find an association between the lowest poverty level and mortality. This is most likely due to a low statistical power as only 2% of the Colombian population were in the lowest level (level V) of the CMPI. The lack of power can be graphically observed by the wide credible intervals of the point estimates. The areas with the lowest poverty index were geographically located far from urban centers, in less developed areas and with low-level health service systems and poorer access to education. In addition, COVID-19 patients from those areas may never made it to a hospital introducing some sort of survival bias in the analysis.

The most important secondary findings of our study were that male sex and increasing age were associated with increased odds of dying in hospitalized COVID-19 patients. This is in line with the current scientific evidence (13, 15, 25). Hu et al. observed a linear association between age and mortality reporting with OR above 12 for age groups above 80-years-of-age (28).

The prevalence of hypertension and chronic kidney disease indicated a protective effect in regard mortality in our data. This is not consistent with previous data but can easily be explained by the study design of our study that does not allow interpretations at the individual level (15, 26, 28–30). Future studies are needed to assess the associations between certain comorbidities in retrospective or prospective cohort studies.

Without a doubt, mortality from COVID-19 was concentrated in the most vulnerable population. Multiple mechanisms have been postulated that may explain why this population had a higher risk of dying during this pandemic. Sharma et al. (31), Hussey et al. (32), and Apenyo et al. (33), mentioned that the marginalized populations during the restrictive public health measures implemented, were forced to mobilize due to economic pressure, which increased the risk of infection and consequently a greater risk of death. It is also believed that the poorest population has a greater burden of comorbidities. For example, in patients with diabetes the presence of the virus leads to an imbalance of the angiotensin-converting enzyme 2 causing an exacerbated inflammatory response. Additionally, inadequate nutrition significantly reduces health, preventing the body from naturally coping with various pathogens. The lack of access to high-complexity healthcare centers and tests that allow prioritization of high-risk cases cause patients to quickly lead to serious conditions or death. Also, the population with a lack of access to public services and with suboptimal housing conditions will be more exposed not only to COVID-19 but also to other infectious diseases, and indeed, to a greater risk of death (14, 31, 33–35). Finally, we observed a gradual reduction in the risk of mortality from COVID-19 from the first wave to the fifth wave, in the highest poverty levels of the CMPI, similar than in the studies by Hussey et al., Leveau et al., and Bastos et al. This may be explained by some increase in herd immunity and vaccination efforts in the population (32, 36). Also, the introduction of new, less severe variants of COVID-19 may played an important role.

The strengths of this study are that it used a poverty index previously validated for the Colombian population. Moreover, this is one of the first ecological studies in the region that determined the associations between poverty measures and hospitalized COVID-19 patients over a 15-month time interval (190,485 patient reports) using existing information from validated, national data sources in Colombia. The high sample size allowed us to further stratify the analysis according to the epidemiological COVID-19 waves that occurred in Colombia. Our study adds important evidence that social disparities are a risk factor for serious health outcomes in a large Latin American population. We hope that the results of this study will motivate further national and international research in this area, so that they will also help as a means for the generation and implementation of public policies in favor of the most vulnerable populations in the face of events with high pandemic potential. Naturally, our study has some limitations. As usual in ecological studies, the data is based on groups rather than individuals. Thus, we do not information on the individual exposure of poverty or chronic disease prevalence. Finally, the adjustment of the model for the different variables assumed a temporal sequence that may not be valid in all cases.

In conclusion, socioeconomic disparities were a major risk factor for mortality in patients hospitalized for COVID-19. Reducing poverty gaps and reducing social inequities continues to be a priority in reducing mortality and other severe complications due to COVID-19 in Colombia. Future research may investigate public health measures and interventions to decrease the poverty gap in order to improve the conditions in the poorest population groups.

## Data availability statement

The original contributions presented in the study are included in the article/Supplementary material, further inquiries can be directed to the corresponding author.

## Ethics statement

The present investigation followed the guidelines of Good Clinical Practice and the guidelines of the Declaration of Helsinki. The analyzes of this research do not have ethical implications since anonymized data was taken from secondary sources of free access. Additionally, according to resolution number 8430 of 1993 of the Colombian Ministry of Health, which establishes the scientific, technical, and administrative standards for health research in the country, this research is classified as risk-free research.

## Author contributions

Data medium: LR and OM. Statistical analysis: OM, CP, and JC. Writing draft preparation, writing, revision, and edition: OM, PD, NB, and BR. Visualization: OM, CP, NB, and PD. Supervision: PD and NB. Project management: PD. All authors contributed to the conceptualization of the research proposal and construction of the final version of the article and approved its submission.
