# Supplementary material for: Socioeconomic disparities associated with mortality in patients hospitalized for COVID-19 in Colombia
Source: Front Public Health. 2023 Mar 29;11:1139379. doi: 10.3389/fpubh.2023.1139379 (PMC10157783; doi:10.3389/fpubh.2023.1139379)
Supplement: Supplementary file 1 [file Data_Sheet_1.pdf]

frontiers

Supplementary Material

1 Supplementary Data

Bayesian multilevel logistic model

$$L = \prod_{i=n}^{n} \text{Bernoulli}\ (y_i|p_i) \qquad \textbf{(1)}$$

$$\text{Ln}\left(\frac{p_i}{1-p_i}\right) = \beta 0 + \beta 1 X1_i + \gamma 0[gi] + \gamma 1[gi]X1_i$$

$$\begin{aligned}
x_i * \beta \\
= \beta_0 + \beta_1\left(\text{Age}^i{}_{[26-50)}\right) + \beta_2\left(\text{Age}^i{}_{[50-75)}\right) + \beta_3\left(\text{Age}^i{}_{[>75]}\right) + \beta_4\left(\text{Sex}^i{}_M\right) \\
+ \beta_5\left(\text{CMPI}^i{}_{[20,40)}\right) + \beta_6\left(\text{CMPI}^i{}_{[40,60)}\right) + \beta_7\left(\mathit{C}\text{MPI}^i{}_{[60,80)}\right) + \beta_8\left(\text{CMPI}^i{}_{[80,100]}\right) \\
+ \beta_9\left(\text{Prevalence of cancer}^i\right) + \ \beta_{10}\left(\text{Prevalence of hypertension}^i\right) \\
+ \beta_{11}\left(\text{Prevalence of diabetes}^i\right) + \beta_{12}\left(\text{Prevalence of chronic kidney disease}^i\right) \\
+ \beta_{13}\left(\text{Population density}^i\right) + \beta_{14}\left(\text{Contagion rate}^i\right)
\end{aligned}$$

$g(i)$: extracts the municipality from the observation i

$$\begin{aligned}
\alpha_i = \gamma_0[g(i)] + \gamma_1\left[g(i)\ \left(\text{CMPI}^i{}_{[20,40)}\right)\right] + \gamma_2\left[g(i)\ \left(\text{CMPI}^i{}_{[40,60)}\right)\right] \\
+ \gamma_3\left[g(i)\ \left(\text{CMPI}^i{}_{[60,80)}\right)\right] + \gamma_4\left[g(i)\ \left(\text{CMPI}^i{}_{[80,100]}\right)\right] \\
+ \gamma_5\left[g(i)\ \left(\text{Prevalence of cancer}^i\right)\right] \\
+ \gamma_6\left[g(i)\ \left(\text{Prevalence of hypertension}^i\right)\right] \\
+ \gamma_7[g(i)\ (\text{Prevalence of diabetes})] \\
+ \gamma_8\left[g(i)\ \left(\text{Prevalence of chronic kidney disease}^i\right)\right] \\
+ \gamma_9\left[g(i)\ \left(\text{Population density}^i\right)\right] + \gamma_{10}\left[g(i)\ \left(\text{Contagion rate}^i\right)\right]
\end{aligned}$$

2 Supplementary Figures and Tables

2.1 Supplementary Figures

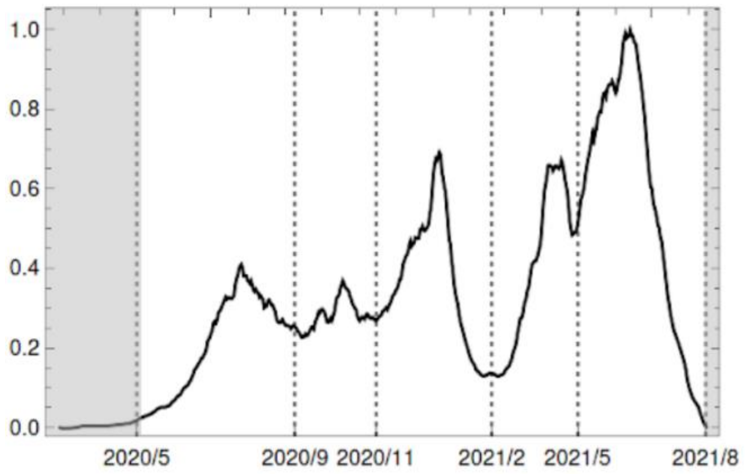

Supplementary Figure 1. Estimation of temporary windows of each wave in the epidemic curve.

To identify the waves in the epidemic curve, the following was carried out: Step 1, a smoothing of the epidemic curve. Step 2, the smoothed series is taken and the difference is estimated. Step 3. Determine the points at which the derivative or difference becomes zero. Five waves were defined (dotted lines in the figure) where in each of them, the Bayesian multilevel logistic model was applied to estimate the OR.

Supplementary Tables

Supplementary Table 1. Wave multilevel logistics models. The ORs were estimated with their respective confidence intervals between the levels of the Colombian Multidimensional Poverty Index (CMPI) adjusted by covariates and the risk of dying when patients are hospitalized for COVID-19.

	Variable	Coefficient	Error	OR	95% CI	
1st wave	**Intercept**	-2.46	0.1	0.09	0.07	0.1
	**Reference category Level I CMPI [0,20]**	1	1	1	1	1
	**Level II CMPI [20,40]**	0.31	0.04	1.37	1.28	1.48
	**Level III CMPI [40,60]**	0.32	0.05	1.37	1.25	1.51
	**Level IV CMPI [60,80]**	0.32	0.1	1.38	1.13	1.68
	**Level V CMPI [80,100]**	0.06	0.25	1.07	0.66	1.82
	**Reference category Age [0,26]**	1	1	1	1	1
	**Age [26,50]**	1.14	0.03	3.14	2.97	3.32
	**Age [50,75]**	2.01	0.03	7.5	7.06	7.96
	**Age [>75]**	2.74	0.03	15.54	14.66	16.49
	**Reference category Sex [Female]**	1	1	1	1	1
	**Sex [Male]**	0.37	0.03	1.44	1.37	1.53
	**Contagion rate**	-0.04	0.39	0.96	0.46	2.11
	**Population density**	0	0	1	1	1
	**Prevalence of hypertension**	-0.05	0.01	0.95	0.94	0.96
	**Prevalence of diabetes**	0.08	0.02	1.08	1.05	1.12

	**Prevalence of chronic kidney disease**	0.02	0.02	1.02	0.99	1.06
	**Prevalence of cancer**	0	0	1	1	1
**2nd wave**	**Intercept**	-4.4	0.11	0.01	0.01	0.02
	**Reference category Level I CMPI [0,20]**	1	1	1	1	1
	**Level II CMPI [20,40]**	0.24	0.04	1.27	1.17	1.37
	**Level III CMPI [40,60]**	0.26	0.06	1.29	1.15	1.45
	**Level IV CMPI [60,80]**	0.25	0.13	1.29	1	1.64
	**Level V CMPI [80,100]**	-0.38	0.46	0.69	0.27	1.62
	**Reference category Age [0,26]**	1	1	1	1	1
	**Age [26,50]**	1.65	0.04	5.22	4.86	5.6
	**Age [50,75]**	2.65	0.04	14.1	13.19	15.12
	**Age [>75]**	3.37	0.03	29.17	27.56	30.92
	**Reference category Sex [Female]**	1	1	1	1	1
	**Sex [Male]**	0.49	0.03	1.64	1.53	1.75
	**Contagion rate**	-0.12	0.42	0.89	0.41	2.04
	**Population density**	0	0	1	1	1
	**Prevalence of hypertension**	-0.04	0.01	0.96	0.95	0.97
	**Prevalence of diabetes**	0.11	0.02	1.12	1.08	1.15
	**Prevalence of chronic kidney disease**	-0.04	0.02	0.96	0.93	1
	**Prevalence of cancer**	0	0	1	1	1
**3rd wave**	**Intercept**	-1.08	0.12	0.34	0.27	0.42
	**Reference category**	1	1	1	1	1

	**Level I CMPI [0,20]**					
	**Level II CMPI [20,40]**	-0.05	0.04	0.95	0.88	1.03
	**Level III CMPI [40,60]**	-0.21	0.07	0.81	0.7	0.93
	**Level IV CMPI [60,80]**	0.09	0.15	1.09	0.82	1.49
	**Level V CMPI [80,100]**	-0.27	0.54	0.76	0.26	2.24
	**Reference category Age [0,26]**	1	1	1	1	1
	**Age [26,50]**	1.11	0.03	3.03	2.84	3.23
	**Age [50,75]**	1.84	0.03	6.31	5.93	6.73
	**Age [>75]**	2.37	0.03	10.69	10.09	11.38
	**Reference category Sex [Female]**	1	1	1	1	1
	**Sex [Male]**	0.34	0.03	1.41	1.32	1.49
	**Contagion rate**	-1.28	0.43	0.28	0.12	0.65
	**Population density**	0	0	1	1	1
	**Prevalence of hypertension**	-0.09	0.01	0.91	0.9	0.92
	**Prevalence of diabetes**	0.09	0.02	1.1	1.06	1.13
	**Prevalence of chronic kidney disease**	0.1	0.02	1.11	1.06	1.15
	**Prevalence of cancer**	0	0	1	1	1
**4th wave**	**Intercept**	-1.5	0.18	0.22	0.16	0.32
	**Reference category Level I CMPI [0,20]**	1	1	1	1	1
	**Level II CMPI [20,40]**	0.06	0.06	1.06	0.95	1.19
	**Level III CMPI [40,60]**	0.1	0.09	1.1	0.92	1.31
	**Level IV CMPI [60,80]**	0.08	0.2	1.08	0.72	1.6

	Level V CMPI [80,100]	1.48	0.5	4.39	1.7	11.51
	Reference category Age [0,26]	1	1	1	1	1
	Age [26,50]	0.99	0.05	2.68	2.45	2.93
	Age [50,75]	1.59	0.05	4.9	4.44	5.4
	Age [>75]	2.24	0.05	9.35	8.48	10.21
	Reference category Sex [Female]	1	1	1	1	1
	Sex [Male]	0.31	0.04	1.37	1.26	1.49
	Contagion rate	-6.3	0.65	0	0	0.01
	Population density	0	0	1	1	1
	Prevalence of hypertension	-0.01	0.01	0.99	0.97	1.01
	Prevalence of diabetes	0.21	0.03	1.24	1.18	1.3
	Prevalence of chronic kidney disease	-0.14	0.03	0.87	0.82	0.93
	Prevalence of cancer	0	0	1	1	1
5th wave	Intercept	-1.41	0.1	0.24	0.2	0.29
	Reference category Level I CMPI [0,20]	1	1	1	1	1
	Level II CMPI [20,40]	0.01	0.03	1.01	0.95	1.08
	Level III CMPI [40,60]	0.09	0.05	1.09	0.99	1.21
	Level IV CMPI [60,80]	0.24	0.11	1.28	1.03	1.59
	Level V CMPI [80,100]	-0.65	0.42	0.52	0.22	1.16
	Reference category Age [0,26]	1	1	1	1	1
	Age [26,50]	0.81	0.03	2.26	2.15	2.38
	Age [50,75]	1.39	0.03	4.02	3.77	4.25
	Age [>75]	1.91	0.03	6.77	6.36	7.23

**Reference category Sex [Female]**	1	1	1	1	1
**Sex [Male]**	0.32	0.02	1.38	1.32	1.45
**Contagion rate**	-1.16	0.38	0.31	0.15	0.64
**Population density**	0	0	1	1	1
**Prevalence of hypertension**	-0.11	0.01	0.9	0.89	0.91
**Prevalence of diabetes**	0.5	0.01	1.65	1.6	1.69
**Prevalence of chronic kidney disease**	-0.28	0.02	0.76	0.73	0.79
**Prevalence of cancer**	0	0	1	1	1
